# Bilateral risk-reducing mastectomy under awake regional anaesthesia with immediate prepectoral reconstruction using lightweight implants in a patient with severe cardiomyopathy: a case report

**DOI:** 10.1093/jscr/rjag336

**Published:** 2026-05-07

**Authors:** Roberto Gennari, Lorenzo Guarrera, Anna Trapani, Virginia Scorsone, Giovanni Misseri

**Affiliations:** Breast Unit, Department of Surgical Oncology, Division of Breast Surgery, Fondazione Istituto G. Giglio, Viale G. Giglio, Cefalù 90015, Italy; European School of Oncology, Via Filippo Turati, 20121, Milano, Italy; Division of Anaesthesia and Intensive Care, Fondazione Istituto G. Giglio, Viale G. Giglio, Cefalù 90015, Italy; Breast Unit, Department of Surgical Oncology, Division of Breast Surgery, Fondazione Istituto G. Giglio, Viale G. Giglio, Cefalù 90015, Italy; Breast Unit, Department of Surgical Oncology, Division of Breast Surgery, Fondazione Istituto G. Giglio, Viale G. Giglio, Cefalù 90015, Italy; Breast Surgery, Campus Bio-Medico University Hospital, Via Alvaro del Portillo 21, Rome 00128, Italy; Division of Anaesthesia and Intensive Care, Fondazione Istituto G. Giglio, Viale G. Giglio, Cefalù 90015, Italy

**Keywords:** risk-reducing mastectomy, awake breast surgery, regional anaesthesia, prepectoral reconstruction, lightweight implants, cardiomyopathy

## Abstract

Risk-reducing mastectomy is an established preventive strategy for carriers of pathogenic germline mutations. In patients with severe cardiomyopathy, general anaesthesia may represent a prohibitive risk. Awake regional anaesthesia may offer a feasible alternative in selected high-risk patients. A 51-year-old woman with a pathogenic ATM mutation and severe hypertrophic cardiomyopathy (NYHA III–IV, ASA IV) underwent bilateral nipple-sparing risk-reducing mastectomy under awake thoracic regional anaesthesia using bilateral erector spinae plane and intertransverse process blocks with conscious sedation. Immediate prepectoral reconstruction with lightweight polyurethane-coated implants was performed. The procedure was completed without complications, recovery was uneventful, and the patient was discharged on postoperative day 1. Awake regional anaesthesia enabled maintenance of spontaneous ventilation and haemodynamic stability, while flap-preserving surgical technique and low-impact reconstruction supported uncomplicated recovery. Tailored perioperative strategies may allow safe completion of risk-reducing breast surgery in carefully selected patients otherwise considered unsuitable for conventional anaesthetic approaches.

## Introduction

Risk-reducing mastectomy is a well-established strategy for individuals carrying pathogenic germline mutations associated with increased breast cancer risk [1]. Immediate implant-based reconstruction is commonly performed; however, severe cardiomyopathy may limit surgical options because general anaesthesia and positive-pressure ventilation can result in significant haemodynamic instability [2].

Awake breast surgery under regional anaesthesia has recently emerged as a feasible option in selected high-risk patients [3–5]. Lightweight breast implants have also been introduced to reduce mechanical stress on soft tissues in reconstructive settings [6]. We present a case illustrating how adaptation of established anaesthetic and reconstructive strategies allowed completion of risk-reducing surgery in a patient otherwise unsuitable for conventional surgical pathways.

**Figure 1:**
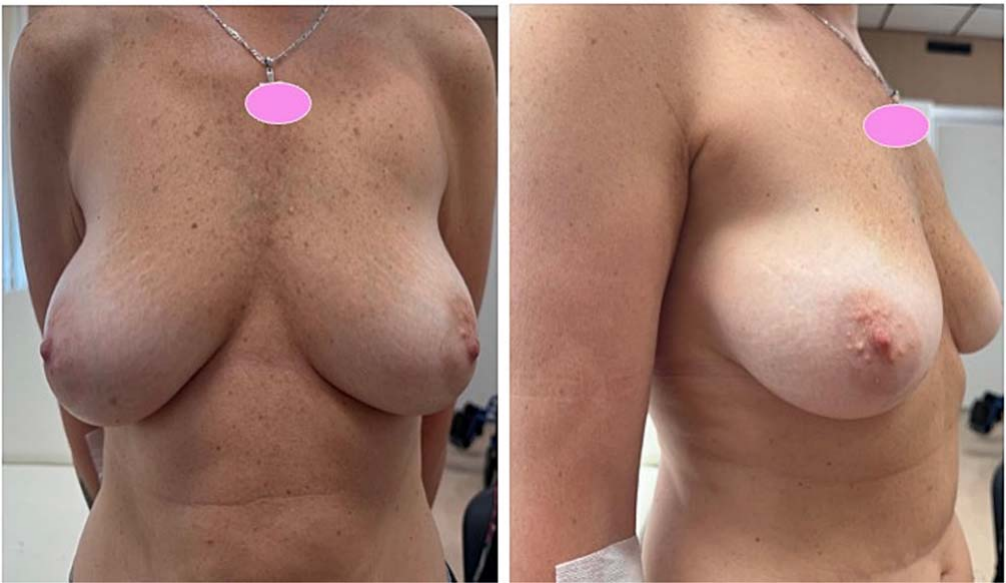
Preoperative frontal and lateral clinical views demonstrating breast ptosis and asymmetry before bilateral risk-reducing mastectomy with immediate implant-based reconstruction.

## Case presentation

A 51-year-old woman carrying a pathogenic ATM mutation was referred for bilateral risk-reducing mastectomy (Fig. 1). Her medical history included severe hypertrophic cardiomyopathy with marked diastolic dysfunction (NYHA class III–IV). She had an implantable cardioverter-defibrillator and was classified as ASA IV.

The patient was undergoing evaluation for heart transplantation, and completion of risk-reducing surgery was required before transplant listing. Following multidisciplinary discussion involving cardiology, anaesthesiology, breast surgery, and plastic surgery, an awake surgical strategy was selected as the safest feasible option.

**Figure 2:**
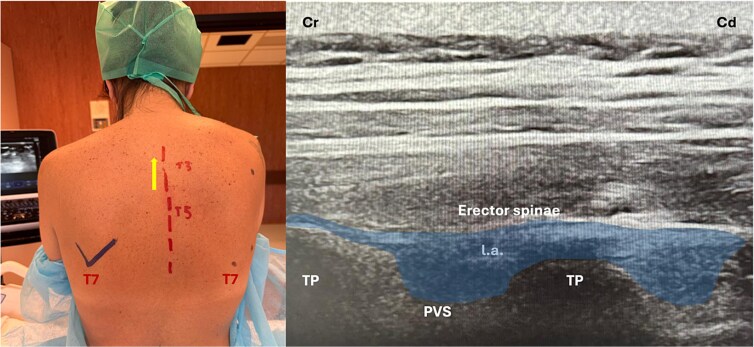
Ultrasound-guided erector spinae plane block at the T3 level showing probe position, anatomical landmarks, and corresponding ultrasound image. TP: transverse process; PVS: paravertebral space; Cr: cranial; Cd: caudal.

Bilateral ultrasound-guided erector spinae plane and intertransverse process blocks were performed at T3 level using ropivacaine with adjuvants (Fig. 2). Sedation consisted of dexmedetomidine infusion with intermittent low-dose ketamine. The patient remained spontaneously breathing and haemodynamically stable throughout the procedure without airway intervention or conversion to general anaesthesia [3–5].

**Figure 3:**
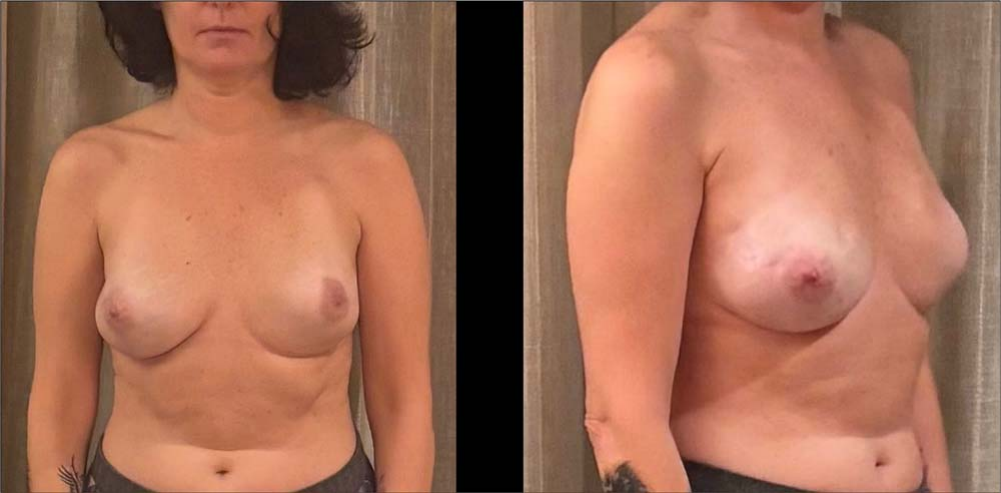
Postoperative frontal and lateral clinical views at 9 months following bilateral risk-reducing mastectomy with immediate prepectoral reconstruction using lightweight implants. A left-sided pacemaker is visible.

Bilateral nipple-sparing mastectomy was performed with meticulous preservation of flap thickness and vascularity. Immediate reconstruction was achieved using a prepectoral approach with polyurethane-coated lightweight implants (B-lite^®^, Polytech Health & Aesthetics GmbH, Dieburg, Germany; 335 ml) (Fig. 3). Total operative time was 120 min without intraoperative complications [7, 8].

Postoperative recovery was uneventful. Adequate analgesia was achieved without opioids, and no cardiovascular or respiratory complications occurred. The patient was discharged on postoperative day 1 and continued the heart transplant evaluation pathway.

## Discussion

Patients with severe cardiomyopathy are often considered unsuitable for elective breast surgery because general anaesthesia and positive-pressure ventilation may compromise haemodynamic stability [2]. In selected cases, awake regional anaesthesia can provide an alternative approach, allowing maintenance of spontaneous ventilation while reducing perioperative physiological stress [3–5].

In the present case, regional anaesthesia combined with light sedation allowed completion of bilateral mastectomy without airway manipulation or opioid use, supporting rapid postoperative recovery [9]. This aligns with growing evidence supporting thoracic regional blocks in breast surgery for high-risk patients [4, 5].

From a surgical perspective, preservation of mastectomy flap vascularity remained central to the reconstructive strategy. The use of a prepectoral approach avoided muscle dissection, while lightweight implants were selected to minimize mechanical load on the soft-tissue envelope [10–12]. These factors likely contributed to the absence of early reconstructive complications [13].

Rather than introducing novel techniques, this case illustrates how careful integration of established anaesthetic and reconstructive principles can expand surgical eligibility in complex patients. Multidisciplinary planning was critical in balancing oncologic necessity with anaesthetic risk.

## Conclusion

Awake regional anaesthesia may allow safe completion of bilateral risk-reducing mastectomy with immediate implant-based reconstruction in carefully selected patients with severe cardiomyopathy. Individualized perioperative planning and low-impact reconstructive strategies were key factors supporting a favourable outcome.
